# Disruption of an RNA-binding hinge region abolishes LHP1-mediated epigenetic repression

**DOI:** 10.1101/gad.305227.117

**Published:** 2017-11-01

**Authors:** Scott Berry, Stefanie Rosa, Martin Howard, Marc Bühler, Caroline Dean

**Affiliations:** 1 John Innes Centre, Norwich NR4 7UH, United Kingdom;; 2 Institute of Biochemistry and Biology, University of Potsdam, DE-14476 Potsdam-Golm, Germany;; 3 Friedrich Miescher Institute for Biomedical Research, 4058 Basel, Switzerland

**Keywords:** chromatin, epigenetics, plant biology, Polycomb, RNA

## Abstract

Epigenetic maintenance of gene repression is essential for development. Polycomb complexes are central to this memory, but many aspects of the underlying mechanism remain unclear. LIKE HETEROCHROMATIN PROTEIN 1 (LHP1) binds Polycomb-deposited H3K27me3 and is required for repression of many Polycomb target genes in *Arabidopsis*. Here we show that LHP1 binds RNA in vitro through the intrinsically disordered hinge region. By independently perturbing the RNA-binding hinge region and H3K27me3 (trimethylation of histone H3 at Lys27) recognition, we found that both facilitate LHP1 localization and H3K27me3 maintenance. Disruption of the RNA-binding hinge region also prevented formation of subnuclear foci, structures potentially important for epigenetic repression.

## Abstract (precis)

In this study, Berry et al. investigated the functions of the different domains of LIKE HETEROCHROMATIN PROTEIN 1 (LHP1) in *Arabidopsis*. They show that LHP1 binds RNA in vitro through the intrinsically disordered hinge region and show that both the hinge region and H3K27me3 recognition facilitate LHP1 localization and H3K27me3 maintenance.

Many genes that regulate differentiation and development in multicellular organisms are targeted for repression by Polycomb-repressive complexes (PRCs). A conserved hallmark of repression by PRC2 is trimethylation of histone H3 at Lys27 (H3K27me3). *Arabidopsis* LIKE HETEROCHROMATIN PROTEIN 1 (LHP1) binds to H3K27me3 in vitro and localizes to repressed Polycomb target genes in vivo (Libault et al. 2005; Turck et al. 2007; Zhang et al. 2007). LHP1 has been found in protein complexes in vivo with the PRC2 component MSI1 (Derkacheva et al. 2013) as well as other plant Polycomb group proteins (Xu and Shen 2008; Wang et al. 2014). It is commonly regarded as a “reader” of H3K27me3 acting downstream from PRC2 activity in the silencing mechanism (Turck et al. 2007). LHP1 has also been found recently to interact with a component of the DNA replication machinery (Zhou et al. 2017). While the role of LHP1 in Polycomb silencing is in contrast to the traditional role of HP1 proteins in the maintenance of heterochromatin, it has been recognized more recently that many eukaryotes contain multiple HP1 paralogs, some of which have euchromatic functions (Canzio et al. 2014). Among the well-studied targets of LHP1 in *Arabidopsis* are key developmental regulators *FLOWERING LOCUS C* (*FLC*) (Mylne et al. 2006; Sung et al. 2006), *FLOWERING LOCUS T* (*FT*), and *AGAMOUS* (*AG*) (Turck et al. 2007).

Like all HP1 proteins, LHP1 contains a chromodomain, which binds methylated histones (Fischle et al. 2003), and a chromoshadow domain, which is involved in protein–protein interactions (Cowieson et al. 2000). These two domains are separated by a less-conserved “hinge” region, which is intrinsically disordered (Keller et al. 2012; Munari et al. 2013). In HP1 proteins, the hinge has been implicated in RNA binding in both *Schizosaccharomyces pombe* (Keller et al. 2012) and mammals (Muchardt et al. 2002) and also has been shown to function in subnuclear localization in *Drosophila* (Smothers and Henikoff 2001). In *S. pombe*, non-sequence-specific RNA binding by HP1^Swi6^ is required for mediating degradation of heterochromatin transcripts (Keller et al. 2012) and the maintenance of appropriate heterochromatin boundaries (Keller et al. 2013). In *Arabidopsis*, LHP1 has been cross-linked in vivo to a long noncoding RNA called *APOLO* (Ariel et al. 2014), suggesting that LHP1 may also bind directly to RNA. However, the functional relevance of this interaction remains unclear.

Here, we show that LHP1 binds RNA in vitro and that the disordered hinge region that mediates this activity is required for LHP1 to localize to and repress Polycomb target genes in vivo. We also show that the subnuclear foci and dynamics of LHP1 are disrupted when the RNA-binding hinge region is perturbed, suggesting a role for LHP1 in generating stable subnuclear structures that facilitate the maintenance of Polycomb target gene repression.

## Results and Discussion

### LHP1 binds RNA in vitro through the hinge region

**Figure 1.**
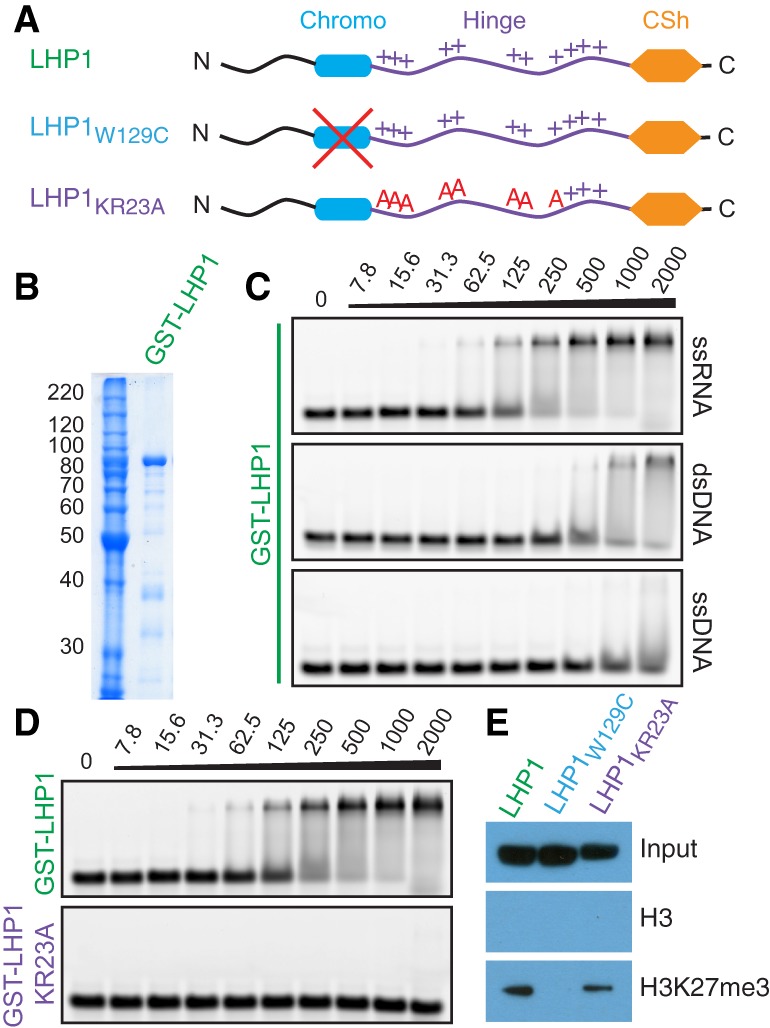
Generation of separation-of-function LHP1 mutants. (*A*) Domain layout of LHP1 and positions of introduced mutations. (+) Basic residues in the hinge region. (*B*) Coomassie-stained SDS-PAGE of purified GST-LHP1 used for EMSAs. Molecular mass is shown in kilodaltons. (*C*) EMSAs with GST-LHP1 and ssRNA, dsDNA, or ssDNA. Protein concentration is shown *above* in nanomolar. (*D*) EMSAs with GST-LHP1 or GST-LHP1_KR23A_ and ssRNA. (*E*) Pull-down assay with peptide from histone H3 residues 21–44 (H3) or the same sequence with H3K27me3. Protein was detected by anti-GST immunoblot.

To determine whether LHP1 is capable of binding RNA, we expressed full-length LHP1 as a glutathione-S-transferase fusion protein in *Escherichia coli* (Fig. 1A,B; Supplemental Figs. S1, S2). We verified that bacterially expressed full-length LHP1 protein was able to recognize H3K9me2-, H3K9me3-, and H3K27me3-modified histone peptides (Supplemental Fig. S1A), in agreement with previous studies of the LHP1 chromodomain (Zhang et al. 2007). Next, using electrophoretic mobility shift assays (EMSAs), we found that purified GST-LHP1 was able to bind to a 40-nucleotide RNA probe in vitro (Fig. 1C). Moreover, these assays revealed that LHP1 had a greater affinity for RNA than for either ssDNA or dsDNA of the equivalent sequence. The apparent equilibrium binding constant (*K*_d_) for RNA was ∼200 nM, which is two orders of magnitude stronger than that previously reported for the LHP1 chromodomain–H3K27me3 interaction (19 µM ± 2 µM) (Zhang et al. 2007).

Previous work on HP1^Swi6^ in *S. pombe* showed that positively charged residues in the intrinsically disordered “hinge” region are important for RNA binding (Keller et al. 2012). We therefore generated mutant LHP1 proteins in which lysine (K) and arginine (R) residues in this region were mutated to alanine (A) (Supplemental Fig. S1B). LHP1_KR9A_, LHP1_KR23A_, and LHP1_KR33A_ represent LHP1 proteins with nine, 23, or 33 such K/R residues mutated. We observed that RNA binding was abolished in LHP1_KR23A_ and LHP1_KR33A_, and reduced in LHP1_KR9A_ (Fig. 1D; Supplemental Fig. S2A,B). These residues are therefore essential for RNA binding by LHP1. Interestingly, previous bioinformatic analysis of LHP1 in divergent plant species also identified these residues as the most conserved sequences outside of the chromodomain and chromoshadow domain (Guan et al. 2011), suggesting a conserved functional role. We also generated a chromodomain LHP1 mutant by mutating a single tryptophan residue in the aromatic cage to cysteine (LHP1_W129C_) (Fischle et al. 2003). Unlike LHP1 and LHP1_KR23A_, which have intact chromodomains, LHP1_W129C_ was unable to recognize H3K27me3 in peptide pull-down assays (Fig. 1E; Supplemental Fig. S2C,D). However, as expected, LHP1_W129C_ retained wild-type RNA-binding activity (Supplemental Fig. S2E).

In summary, LHP1 can bind to RNA in vitro, and this requires evolutionarily conserved positively charged K/R residues in the “hinge” region. Furthermore, LHP1_KR23A_ and LHP1_W129C_ represent separation-of-function mutants of LHP1, which selectively disrupt RNA binding and H3K27me3 binding, respectively.

### The LHP1 RNA-binding region is required for Polycomb target gene repression in vivo

To determine the functional requirement for RNA binding and H3K27me3 binding by LHP1 in vivo, we generated plants expressing LHP1, LHP1_KR23A_, and LHP1_W129C_ as eGFP fusion proteins in a genetic background that lacks functional *LHP1* (*FRI lhp1-6*). Transgenes included the entire *LHP1* genomic sequence from 2.5 kb upstream of to 1.1 kb downstream from the coding sequence (Supplemental Fig. S3A). Since mutation of basic “hinge” residues in LHP1_KR23A_ perturbs a nuclear localization signal (NLS) (Gaudin et al. 2001), we found that it was necessary to introduce an exogenous C-terminal NLS to ensure that LHP1_KR23A_ was targeted to the nucleus (LHP1_KR23A_-NLS) (Supplemental Fig. S3B). To minimize reintroduction of basic residues, we used an atypical NLS (SVLGKRKFA) (Kosugi et al. 2008). We observed limited phenotypic variability between independent transgenic lines for each construct (Supplemental Fig. S4A). Moreover, we verified that mRNA splicing was unaffected by introduction of mutations (Supplemental Fig. S4B–E), that expression of transgenic *LHP1* was similar to endogenous *LHP1* levels in wild-type plants (Supplemental Fig. S5A), and that mutated proteins were present at similar levels (Supplemental Fig. S5B).

**Figure 2.**
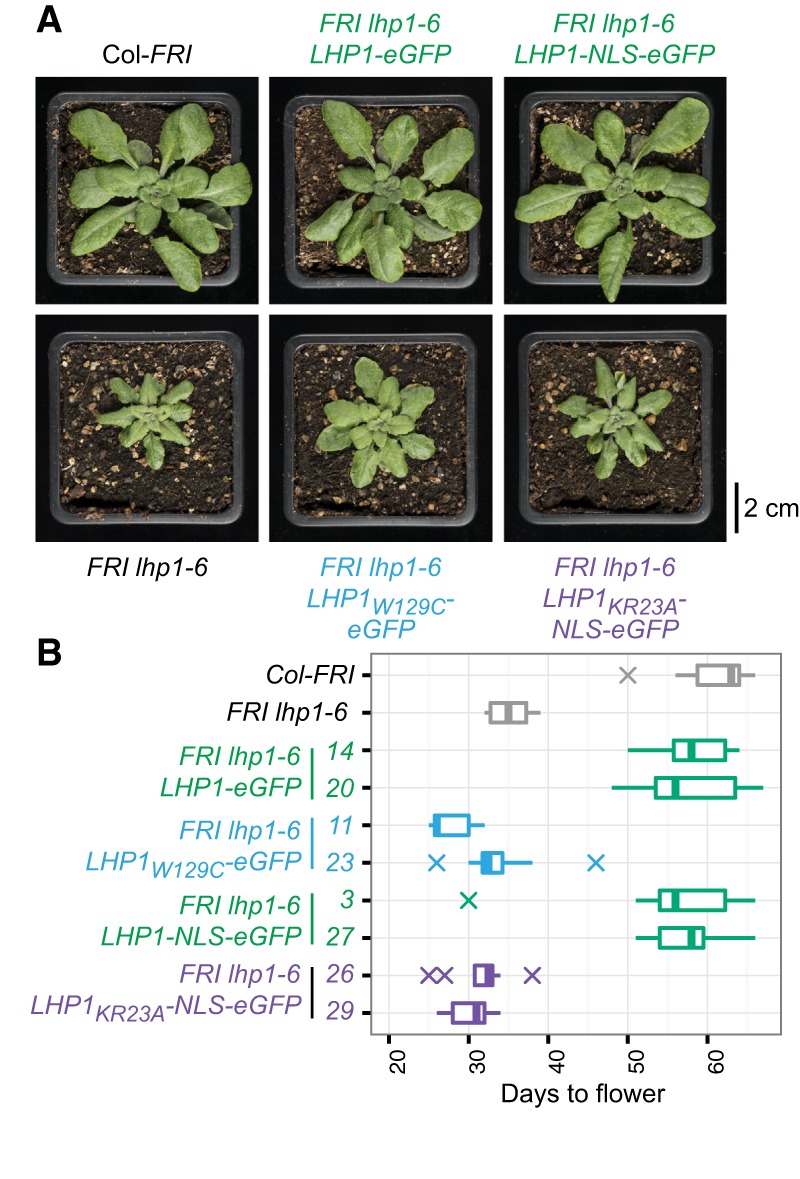
Phenotypes of plants expressing RNA-binding (KR23A) or chromodomain (W129C) *LHP1* mutants. (*A*) Rosette morphology for wild-type (Col-*FRI*), *FRI lhp1-6*, or *FRI lhp1-6* plants expressing the indicated LHP1-eGFP fusion protein. (*B*) Flowering time (measured in days after sowing) for plants grown at 20°C in long day (16-h day/8-h night) conditions. Two independent transgenic lines are shown for each *LHP1* construct.

*lhp1* mutants flower early due to their inability to repress *FT* (Kotake et al. 2003), a positive regulator of the floral transition. Moreover, they show small plant size and downward-curled rosette leaves (Fig. 2A), which may be related to overexpression of *AG*, *APETALA3* (*AP3*), or other Polycomb target genes (Krizek and Meyerowitz 1996; Kotake et al. 2003). We observed complementation of these three phenotypes when *lhp1-6* plants were transformed with wild-type *LHP1-eGFP* or *LHP1-NLS-eGFP* (Fig. 2), indicating that neither eGFP nor NLS-eGFP interfered with LHP1 function in vivo. However, we observed that plants expressing *LHP1_W129C_-eGFP* and *LHP1_KR23A_-NLS-eGFP* remained early flowering, similar to *FRI lhp1-6* (Fig. 2B; Supplemental Fig. S5C). The small size and curled leaf phenotypes of *FRI lhp1* were partially rescued with *LHP1*_*W129C*_. However, the morphology of *LHP1_KR23A_-NLS* plants was indistinguishable from the parental line *FRI lhp1-6* (Fig. 2A). Together, these results suggest that *LHP1_KR23A_-NLS* is a complete loss-of-function *LHP1* mutation, while *LHP1*_*W129C*_ is partially functional.

**Figure 3.**
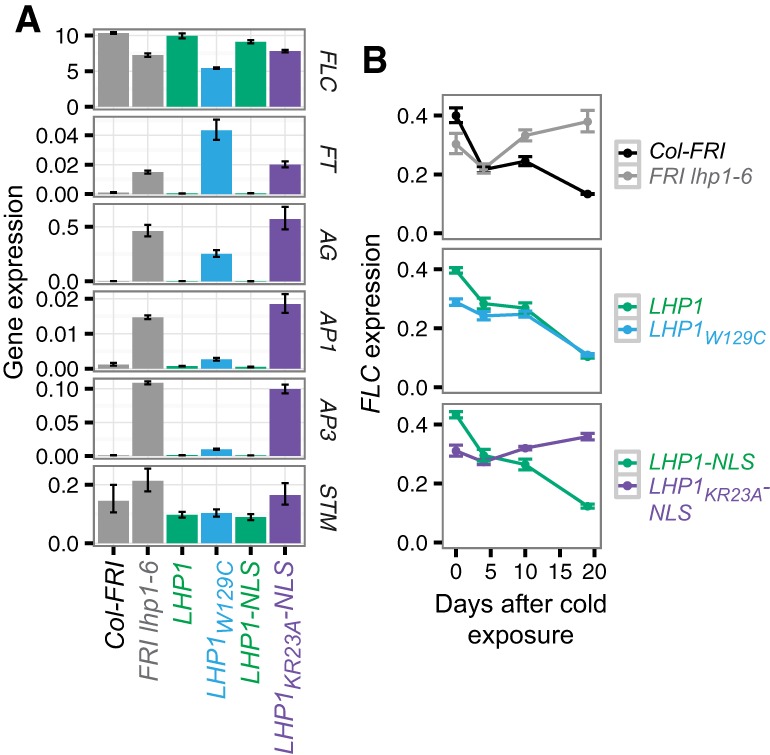
Separation-of-function *LHP1* mutations differentially affect Polycomb target gene expression. (*A*) Gene expression quantified by RT-qPCR in 10-d-old seedlings. Data were normalized to *UBC*. (*B*) Time course of *FLC* expression after a 4-wk cold treatment. Data were normalized to *UBC* and then expressed relative to *FLC* expression without cold. In both cases, error bars represent SEM for three samples each of three independent lines. *n* = 9 for transgenic lines; *n* = 3 for Col-*FRI* and *FRI lhp1-6*.

To investigate this in more detail, we profiled expression of Polycomb target genes *FLC*, *FT*, *AG*, *AP1*, *AP3*, and *SHOOT MERISTEMLESS* (*STM*) by quantitative PCR (qPCR). In all cases, we found that *LHP1* or *LHP1-NLS* plants showed gene expression similar to that of wild-type Col-*FRI* plants, whereas *LHP1_KR23A_-NLS* was similar to the parental *lhp1* mutant (Fig. 3A). The chromodomain mutant *LHP1*_*W129C*_ showed a more complex gene expression profile: While *FT* was not repressed in *LHP1*_*W129C*_, other Polycomb target genes such as *AG*, *AP1*, and *AP3* showed reduced expression compared with *lhp1-6* or *LHP1_KR23A_-NLS*.

To further investigate how these mutations affect LHP1 function, we turned to *FLC*, a well-characterized locus subject to chromatin-based epigenetic memory (Berry and Dean 2015). *FLC* is repressed during prolonged cold exposure, and repression is maintained in *cis* by a Polycomb-based epigenetic memory after cold (Berry et al. 2015) that depends on LHP1 (Mylne et al. 2006; Sung et al. 2006). We found that plants expressing *LHP1_KR23A_-NLS-eGFP* were unable to maintain *FLC* repression after cold exposure, analogous to *lhp1-6* mutants (Fig. 3B). In contrast, although warm-grown *LHP1_W129C_* seedlings showed reduced *FLC* expression compared with wild type (Fig. 3A), maintenance of *FLC* repression after cold was not affected by *LHP1*_*W129C*_ (Fig. 3B). Like many chromodomain proteins, it has long been assumed that LHP1 acts as a histone “reader”—recognizing its targets by binding methylated histones, providing a feedback mechanism to reinforce H3K27me3 levels (Turck et al. 2007; Derkacheva et al. 2016). Indeed, previous work showed that mutation of W129 to CCER results in loss of LHP1 function (Exner et al. 2009). The observation that epigenetic silencing of *FLC* is unperturbed in *LHP1*_*W129C*_ (which has reduced affinity for H3K27me3) (Fig. 1E; Supplemental Fig. S2D) suggests that this feedback mechanism is not absolutely required for long-term silencing at *FLC*. Alternatively, LHP1_W129C_ may still be conferring sufficient binding to H3K27me3 to maintain *FLC* silencing, as compared with the loss-of-function CCER mutation.

In summary, phenotypic and gene expression data indicate that the RNA-binding hinge region of LHP1 performs an important function in vivo, without which LHP1 is unable to repress Polycomb target genes. Conversely, the ability of LHP1 to recognize H3K27me3 appears to be dispensable at some Polycomb targets, including *FLC*.

### RNA-binding mutant LHP1_*KR23A*_ does not associate with Polycomb target genes

In *S. pombe*, RNA binding by HP1^Swi6^ was reported to act downstream from chromatin targeting and H3K9 methylation to mediate the degradation of RNA transcribed from within heterochromatin (Keller et al. 2012). We therefore wondered whether LHP1_KR23A_-NLS is able to localize to Polycomb target genes such as *FLC* but was simply unable to have a repressive effect. We therefore performed chromatin immunoprecipitation (ChIP) experiments to analyze both LHP1 binding and H3K27me3 levels at *FLC* during and after cold exposure.

**Figure 4.**
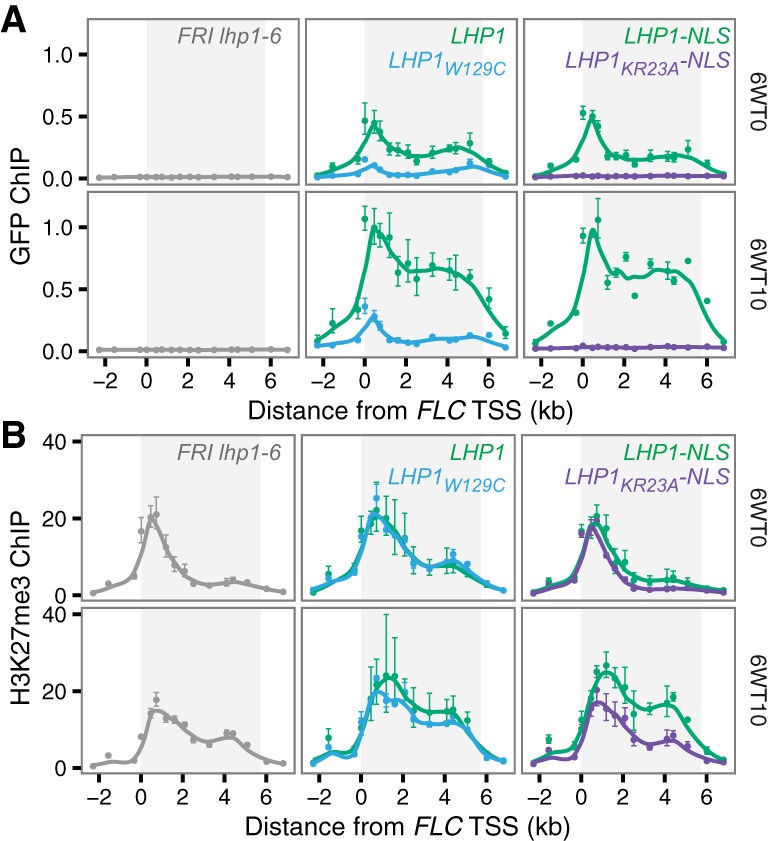
*FLC* chromatin during and after cold treatment in separation-of-function *LHP1* mutants. (*A*) LHP1 occupancy at *FLC* as determined by anti-GFP ChIP-qPCR. Data are represented as percentage of input DNA. (6WT0) The end of 6 wk of cold treatment; (6WT10) 10 d after a 6-wk cold treatment. Data for wild-type LHP1-eGFP were described previously (Yang et al. 2017). (*B*) H3K27me3 ChIP-qPCR represented as a percentage of H3 ChIP. In both *A* and *B*, error bars represent SEM. *n* = 3. The line was obtained by LOESS smoothing.

Silencing of *FLC* during cold exposure is accompanied by deposition of H3K27me3 at a small region downstream from the transcription start site, known as the “nucleation region” (Angel et al. 2011). In wild-type plants, H3K27me3 and LHP1 subsequently “spread” to cover the entire locus in the weeks after cold. It is “spreading” rather than “nucleation” that is perturbed in *lhp1* mutants (Yang et al. 2017). We observed that wild-type LHP1 and LHP1-NLS both localized to the *FLC* nucleation region during cold and subsequently increased and spread across the entire locus after cold (Fig. 4A). We detected qualitatively similar but quantitatively reduced binding for LHP1_W129C_. However, we found that LHP1_KR23A_-NLS did not bind to *FLC* either during or after cold. The reduction in ChIP signal observed for LHP1_W129C_ could be due to a higher turnover of LHP1_W129C_ on chromatin, resulting in a lower probability of cross-linking during our ChIP protocol. Alternatively, a subpopulation of loci may lose binding altogether in this mutant. In the latter case, however, we would expect to see somewhat elevated *FLC* expression in *LHP1*_*W129C*_ plants after cold exposure, which we did not observe (Fig. 3B).

Consistent with our expression data, we found that nucleation of H3K27me3 during cold and spreading across the locus after cold were similar in *LHP1*, *LHP1-NLS*, and *LHP1*_*W129C*_ plants but that spreading was compromised in *LHP1_KR23A_-NLS*, similar to the complete loss-of-function *lhp1* mutant (Fig. 4B). At other Polycomb target genes (*STM*, *AG*, and *FT*), LHP1_KR23A_-NLS showed no association, and LHP1_W129C_ showed low levels, with H3K27me3 accumulation compromised in both cases (Supplemental Fig. S6).

These data demonstrate that localization of LHP1_KR23A_-NLS to the chromatin of *FLC* and other Polycomb target genes is compromised, which leads to a failure to maintain H3K27me3 and elevated gene expression.

### The hinge region of LHP1 is required for formation of subnuclear foci

**Figure 5.**
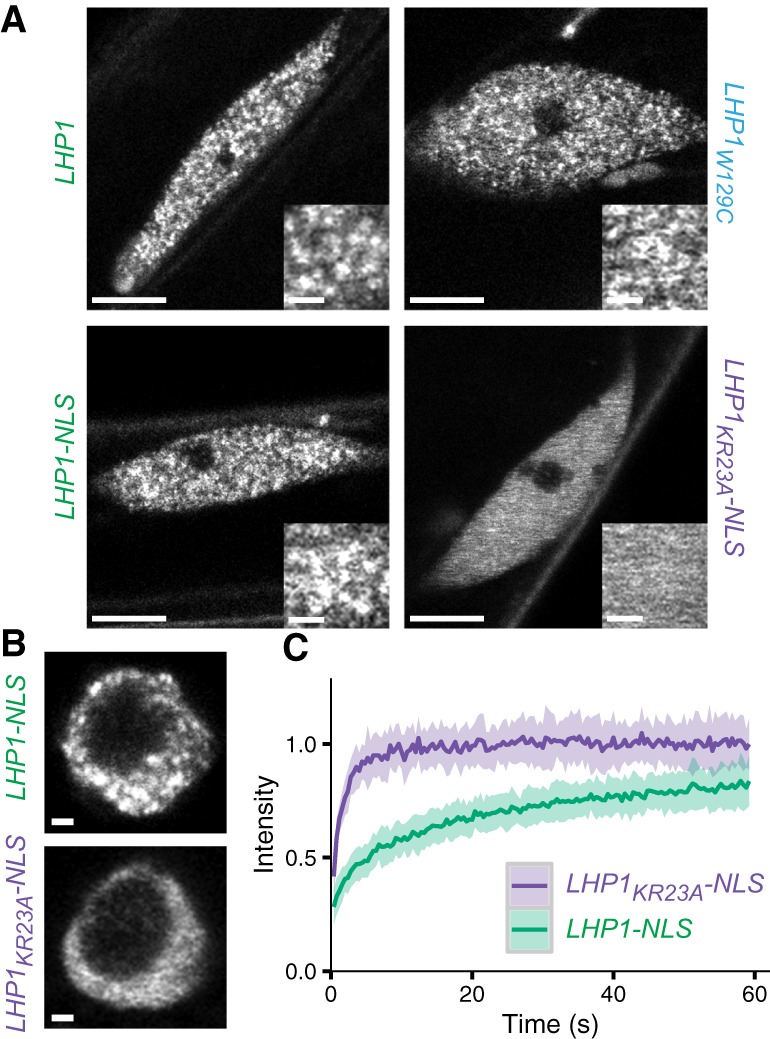
KR23A mutation alters subnuclear distribution and dynamics of LHP1. (*A*) Subnuclear distribution of LHP1-eGFP fusion proteins in differentiated root epidermal cells. *Inset* panels show a magnified view. Bars: all except for *insets*, 5 µm; *insets*, 1 µm. (*B*) Subnuclear distribution in meristematic root epidermal cells. Bars, 1 µm. (*C*) Fluorescence recovery after photobleaching (FRAP) recovery curves (mean ± SD) in root epidermal cells. *n* = 22 LHP1-NLS-eGFP; *n* = 18 LHP1_KR23A_-NLS-eGFP. (*A*–*C*) All data are from 7-d-old seedlings.

To determine how perturbation of the RNA-binding hinge region and H3K27me3-binding chromodomain independently affects the subnuclear distribution of LHP1, we acquired high-resolution images of LHP1-eGFP separation-of-function mutants in 7-d-old root epidermal nuclei. We observed that LHP1-eGFP exhibited a punctate nuclear distribution (Fig. 5A; Supplemental Fig. S7A) reminiscent of Polycomb bodies (Pirrotta and Li 2012). Subnuclear foci have been observed previously in differentiated cells when LHP1-GFP was expressed from a *35S* promoter (Gaudin et al. 2001; Libault et al. 2005). At endogenous levels, we observed that LHP1-eGFP formed foci in both differentiated and meristematic cells (Fig. 5B; Supplemental Fig. S7B). The size of foci appeared similar between all cell types (0.2–0.4 µm); however, this was close to the theoretical diffraction limit of our imaging setup (174 nm). Foci were not dramatically disrupted by the W129C chromodomain mutation; however, they were abolished by the KR23A mutation, with LHP1_KR23A_-NLS-eGFP exhibiting a diffuse nuclear staining pattern (Fig. 5A,B; Supplemental Fig. S7A,B).

To analyze the dynamic behavior of these proteins, we performed fluorescence recovery after photobleaching (FRAP) experiments in 7-d-old seedlings. We verified that recovery times were similar for LHP1-eGFP, LHP1_W129C_-eGFP, and LHP1-NLS-eGFP (Supplemental Fig. S7D,E) but found that the recovery dynamics of the KR23A mutant were significantly faster as compared with LHP1-NLS-eGFP (Fig. 5C; Supplemental Fig. S7C). This indicates that sequestering LHP1 in foci slows FRAP recovery.

The RNA-binding hinge region of LHP1 is predicted to be intrinsically disordered, similar to other HP1 proteins (Supplemental Fig. S1; Keller et al. 2012). Such regions can undergo phase transitions to form non-membrane-bound subcellular compartments, frequently containing RNA (Lin et al. 2015). It is interesting to speculate that the subnuclear foci that we observed may therefore represent a phase-separated compartment, as observed recently for mammalian HP1α (Larson et al. 2017) and *Drosophila* HP1a (Strom et al. 2017). RNA-binding to the LHP1 hinge region could precipitate formation of nuclear LHP1 bodies to maintain a locally high concentration of LHP1 at Polycomb target genes. The relatively weak H3K27me3–LHP1 interaction may further enhance association of chromatin with the silencing compartment, explaining why H3K27me3 recognition is functionally critical in some cases (e.g. *FT*) (Fig. 3; Supplemental Fig. S6) but not others (e.g., *FLC*).

An LHP1–RNA interaction has been shown to enhance a chromatin loop at the *PINOID* locus (Ariel et al. 2014). At *FLC*, a gene loop links 5′ and 3′ regions, but this is disrupted early during cold exposure and so is unlikely to be involved in LHP1 function in the maintenance of *FLC* silencing (Crevillen et al. 2012). High transcription levels are thought to antagonize Polycomb silencing (Berry et al. 2017), so nascent transcripts will be rare in the PRC2 silenced state. Nonetheless, since transcription initiation and elongation rates are correlated (Wu et al. 2016), any RNA produced from infrequent transcription events at a repressed locus would be associated in *cis* for appreciable durations. Alternatively, LHP1-dependent repressive chromatin domains may form through protein aggregation with non-sequence-specific RNAs to provide relatively stable maintenance of repression at many Polycomb targets (Keller et al. 2012; Stunnenberg et al. 2015). Further work will be needed to examine the interplay between chromatin-based epigenetic repression, RNA, and genome organization.

## Materials and methods

### Protein expression and purification

*E. coli* BL21 Rosetta (DE3) pLysS cells (Novagen) were transformed with pGEX-LHP1 (plasmid details are in the Supplemental Material) and incubated for 16 h at 37°C on LB medium plates containing selective antibiotics. One liter of cultures was grown until OD_600_ = 0.7 for 4–8 h at 37°C and then induced at 20°C with 0.25 mM isopropyl β-D-1-thiogalactopyranoside (IPTG). After 16 h, cells were collected by centrifugation, washed in PBS, and frozen in liquid nitrogen. Cell pellets were resuspended in 5 mL of cold lysis buffer (25 mM Tris-HCl at pH 7.5, 500 mM NaCl, 1% [w/v] Triton X-100, Complete protease inhibitor cocktail [Roche]) per gram of cells and sonicated on ice using a Branson sonifier (10 times for 30 sec at 30%–40% duty). LHP1 was purified from cleared lysate using 1–2 mL of glutathione-sepharose 4B resin (GE Healthcare) by gravity flow according to the manufacturer's instructions. Washes were performed with 25 mM Tris-HCl (pH 7.5), 500 mM NaCl, 0.1% (w/v) Triton X-100, 25 mM Tris-HCl (pH 8.0), and 100 mM NaCl. GST fusion protein was eluted in 25 mM Tris-HCl (pH 8.0), 100 mM NaCl, and 50 mM reduced L-glutathione (Sigma-Aldrich, G4251).

For peptide pull-down assays, pooled fractions were concentrated and exchanged into 50 mM Tris (pH 7.5), 150 mM NaCl, 0.1% (w/v) NP-40, and 1 mM DL-dithiothreitol (DTT) (Sigma, D9779) using centrifugal concentrators (Amicon). For EMSA, proteins were further purified by anion exchange chromatography. Specifically, pooled eluates were loaded on a 5-mL HiTrap Q FF column (GE Healthcare) using an ÄKTA fast protein liquid chromatographer (FPLC) (GE Healthcare) at 4°C. Proteins were eluted in fractions using a 0%–70% gradient of buffer QA (25 mM Tris at pH 7.5, 100 mM NaCl, 1 mM DTT, 0.5 mM EDTA) and buffer QB (QA with 1 M NaCl). Fractions containing GST-LHP1 were identified by UV absorbance and SDS-PAGE, pooled, and exchanged into buffer QA.

### EMSA

ssRNA and ssDNA probes used for EMSA had the sequence Cy5-CUC CUCCGGCGAUAAGUACGCCUUUUCCUUACCUGGGUUU, with U exchanged for T in DNA probes (derived from FLC exon 1/intron 1). Probes were synthesiszd and purified by high-performance liquid chromatography (HPLC) (Integrated DNA Technologies). The dsDNA probe was generated by annealing a complementary unlabeled oligonucleotide to the labeled ssDNA probe.

Concentrated proteins were diluted to 10 μM in QA buffer and then diluted further to 2 μM in EMSA buffer (20 mM HEPES-KOH at pH 7.5, 100 mM KCl, 0.05% NP-40). One microliter of 100 nM Cy5-labeled RNA or DNA (Integrated DNA Technologies) was added to 9 μL of protein for 30 min at room temperature. Four microliters of 50% glycerol was added immediately before loading on an RNase-free 1.6% TBE-agarose gel. After electrophoresis, probes were visualized using a Typhoon 9400 gel scanner (GE Healthcare).

### Peptide pull-down assay

Peptides from histone H3 residues 21–44 (ATKAARSAPATGGVKK PHRYRPG-GK-Biotin) were either unmodified (Anaspec, 64440) or carried K27me3 (Anaspec, 64367) or K36me3 (Anaspec, 64441) modifications. Peptides from histone H3 residues 1–21 (ARTKQTARKSTGG KAPRKQLA-GGK-Biotin) were either unmodified (Anaspec, 61702) or carried K4me2 (Anaspec, 64356), K4me3 (Anaspec, 64357), K9me2 (Anaspec, 64359), or K9me3 (Anaspec, 64360). Biotinylated peptide (0.5 μg) was incubated for 15 min at 4°C in binding buffer (50 mM Tris at pH 7.5, 150 mM NaCl, 0.1% [w/v] NP-40, 1mM DTT) with 7 μg of washed streptavidin Dynabeads (MyOne T1; Invitrogen, 65601). After washing beads to remove unbound peptides, GST fusion proteins (5 μM in binding buffer) were added for 15 min at 4°C. After washing, bound proteins were denatured in Laemmli buffer at 90°C, separated by SDS-PAGE, and detected by immunoblot using an anti-GST antibody (Abcam, ab92).

### Plant materials

*FRI*^Sf2^ *lhp1-6* was generated by crossing *lhp1-6* (SALK_011762) with Col-*FRI*^Sf2^. *LHP1-eGFP* plasmids (details are in the Supplemental Material) were transformed into *FRI*^Sf2^ *lhp1-6* using *Agrobacterium tumefaciens*. Forty-eight independent transgenic lines were selected for each construct. Five lines with flowering time closest to the median for each construct were chosen for further propagation. After verifying by segregation ratio that these contained a single transgene insertion site, two lines with transgenic *LHP1* expression most similar to that of endogenous *LHP1* were used for all remaining experiments. Correct splicing of transgenic *LHP1* mRNA was verified by RT–PCR, and mutations were verified by sequencing cDNA (Supplemental Fig. S4). Wild-type LHP1-eGFP lines were published previously (Yang et al. 2017).

### Gene expression analysis

RNA extraction and qPCR were performed as described previously (Berry et al. 2015).

### ChIP

ChIP experiments were performed as described previously (Yang et al. 2017). Anti-GFP (Abcam, ab290) was used with Protein A agarose/salmon sperm DNA (Millipore, 16-157). Anti-H3 (Abcam, ab1791) or anti-H3K27me3 (Millipore, 07-449) was used with protein A Dynabeads (Invitrogen). qPCR primers are listed in Supplemental Table S1.

### Microscopy

Seedlings were grown for 7 d at 22°C with 16 h of light in vertically orientated Petri dishes containing Murashige and Skoog medium minus glucose. The 7-d-old seedlings were mounted in water between a slide and coverslip for imaging. Single optical sections of root nuclei were captured with a Zeiss LSM 710 microscope using a plan-apochromat 40×/1.4 oil objective and 488-nm excitation laser. Signal was detected at 500–550 nm. Images were collected with a pinhole size of 1 airy at 1024 × 1024-pixel density, imaging zoom 4, pixel dwell time 1.58 µsec, and four-line averaging. Images were processed with ImageJ.

### FRAP

Seven-day-old seedlings were grown and imaged on a coverglass chamber system (Lab-Tek II, 155360). Seedlings were grown for 7 d at 22°C with 16 h of light in vertical orientation. FRAP was performed on a Zeiss LSM 710 inverted laser confocal microscope using an EC plan-neofluar 40× objective/NA 1.3 (195-μm pinhole, imaging zoom 7). A prescan was acquired followed by five bleaching pulses (speed 200–500 pixels/msec) using 100% laser power (488 nm) and a bleach area of ∼1 µm in diameter. Single-plane images (256 × 256 pixels) were collected every 0.4 sec with bidirectional scanning and a pixel dwell time of 2.55 µsec. After background subtraction, FRAP recovery curves were normalized for the loss of fluorescence due to imaging and bleach pulse (double normalization) (Phair et al. 2004).

## Supplementary Material

Supplemental Material
