# Supplementary material for: Disruption of an RNA-binding hinge region abolishes LHP1-mediated epigenetic repression
Source: Genes Dev. 2017 Nov 1;31(21):2115–20. doi: 10.1101/gad.305227.117 (PMC5749160; doi:10.1101/gad.305227.117)
Supplement: Supplemental Material [file supp_31_21_2115__index.html]

Disruption of an RNA-binding hinge region abolishes LHP1-mediated epigenetic repression — Supplemental Material 

# Disruption of an RNA-binding hinge region abolishes LHP1-mediated epigenetic repression

## Supplemental Material

- Supplemental\_Materials.pdf
