# Supplementary material for: Disruption of an RNA-binding hinge region abolishes LHP1-mediated epigenetic repression
Source: Genes Dev. 2017 Nov 1;31(21):2115–20. doi: 10.1101/gad.305227.117 (PMC5749160; doi:10.1101/gad.305227.117)
Supplement: Supplemental Material [file supp_gad.305227.117_Supplemental_Materials.pdf]

SUPPLEMENTAL METHODS

Constructs for bacterial protein expression

LHP1 was amplified by PCR from Arabidopsis cDNA (Col-0) and cloned into pGEX-4T-1 (GE Healthcare) to generate an N-terminal glutathione-S-transferase (GST)-LHP1 fusion. Site-directed mutagenesis of this construct was used to generate GST-LHP1$_{W129C}$, and also to introduce a synonymous SexAI site (+489 from ATG). LHP1$_{KR9A}$, LHP1$_{KR23A}$ and LHP1$_{KR33A}$ 'hinge' fragments were synthesized (GeneStrings, Invitrogen) and subcloned into pGEX-LHP1 using SexAI/AfeI.

Generating LHP1-eGFP constructs for *in vivo* expression

Genomic *LHP1* sequence from -2406 to +3164 bp (relative to ATG) was synthesized in 3 distinct modules: upstream/5'UTR; CDS; 3'UTR/downstream (Invitrogen), which were assembled using Golden Gate modular cloning. All endogeneous BsaI and BpiI sites were mutated in synthesized sequences and a synonymous G to A mutation was included in exon 4 (+1015 bp) to create a SexAI site for subcloning mutated LHP1 hinge domain fragments (GeneStrings, Invitrogen). Site-directed mutagenesis was used to generate the W129C mutation, and to insert nuclear-localisation sequences. Assembled LHP1 constructs were transferred to a custom-made gateway donor plasmid (pL2V-GW-EXPORT2) containing Gateway attL1 and attL2 sites flanking the Golden Gate assembly product. L/R reaction (Invitrogen) was then used to transfer the LHP1 construct to the binary plasmid pSLJ-DEST (based on pSLJ755I6 (Jones et al. 1992)).

Microscopy

Initial studies of subcellular localisation were performed on a Leica TCS SP5 confocal microscope using a 20x/0.7 water objective and Leica HyD hybrid detectors. With 458 nm excitation laser, signal was detected at 500-550 nm. Channel images were combined and rescaled using ImageJ.

SUPPLEMENTAL FIGURES

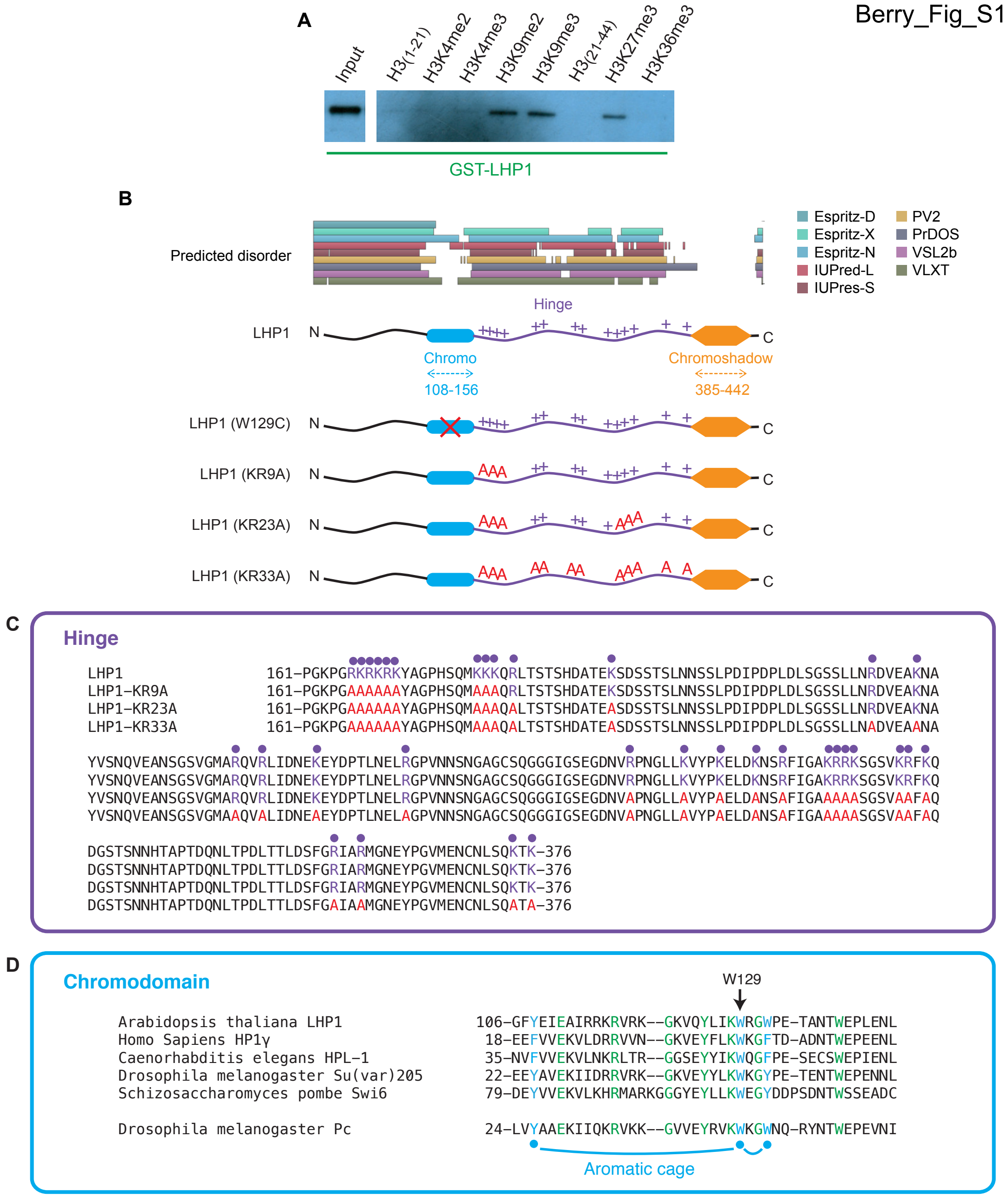

Supplemental Figure S1. Design of LHP1 mutants. (A) Peptide pulldown assay with histone H3 peptides and GST-LHP1. (B) Predicted disorder of LHP1 using several algorithms shown in legend (D^2P^2, http://d2p2.pro), domain layout and positions of introduced mutations. '+' indicates basic residues in the hinge region. (C) Precise positions of mutations in $LHP1_{KR9A}$, $LHP1_{KR23A}$, $LHP1_{KR33A}$ mutants. (D) Alignment of chromodomain sequences between LHP1 and several HP1 homologues, highlighting the key residues which form the aromatic cage required for recognition of methylated H3 (Fischle et al. 2003). Also shown is the Drosophila Polycomb (Pc) chromodomain.

Berry_Fig_S2

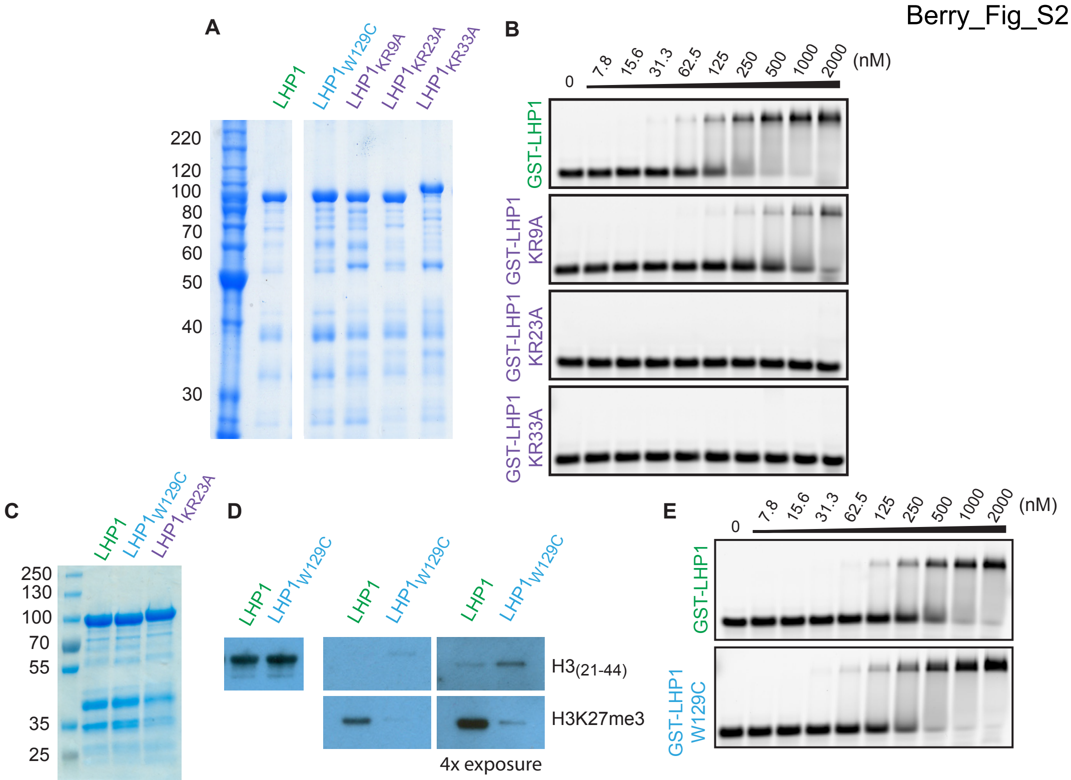

Supplemental Figure S2. Characterisation of *in vitro* activity of LHP1 mutants. (A) Purified proteins used for EMSA. (B) EMSA with GST fusion protein indicated on left and single-stranded RNA (ssRNA). Protein concentration in nM. (C) Purified proteins used in peptide pulldown assay (Figure 1E). (D) Repeat of peptide pulldown assay with LHP1 and LHP1$_{W129C}$. Left panel shows input, right panels show pulldown at two different exposures (E) EMSA with GST fusion protein indicated on left and single-stranded RNA (ssRNA).

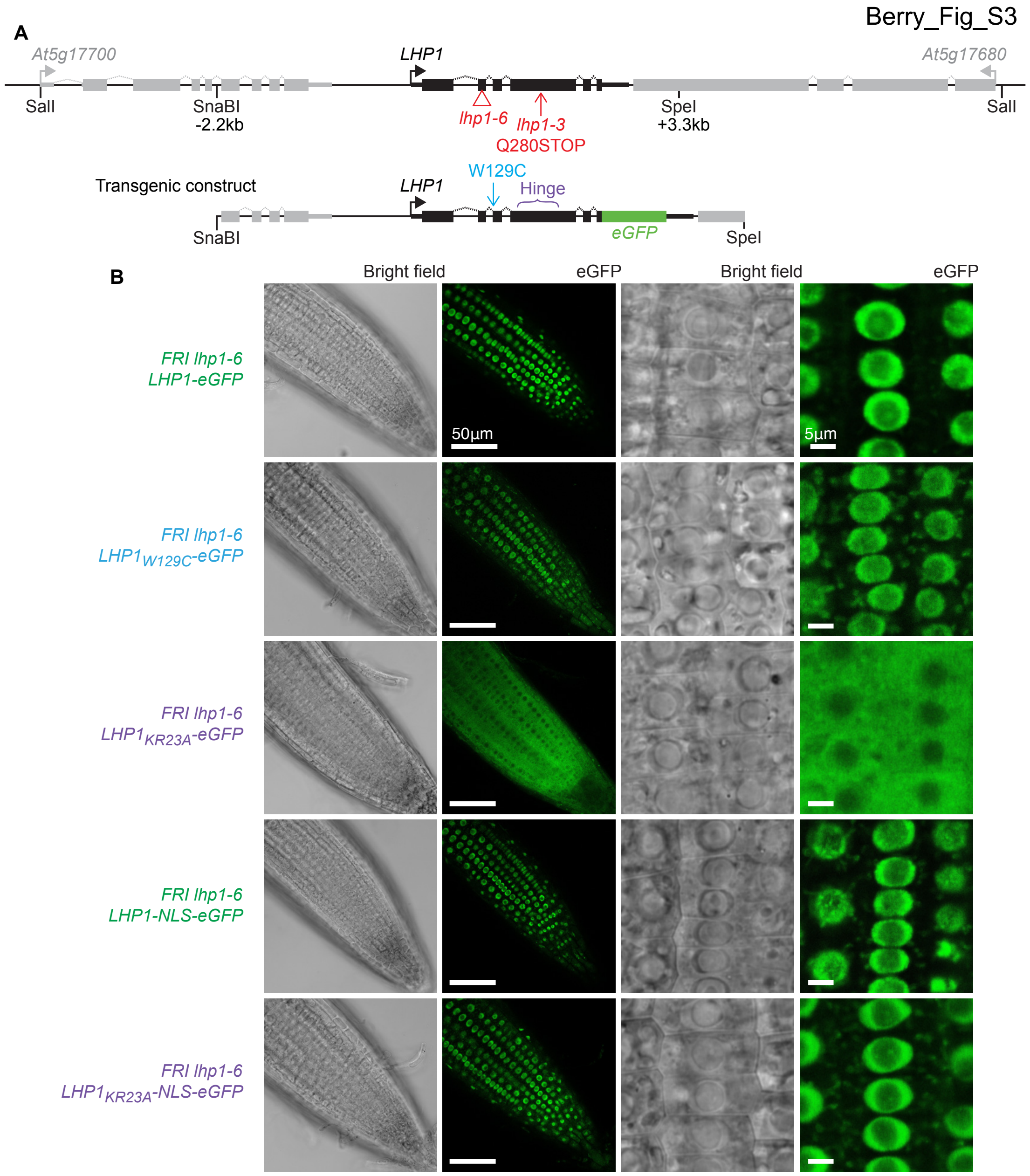

Supplemental Figure S3. Generation of transgenic *LHP1* plants. (A) Genomic *LHP1* locus indicating upstream and downstream genes and positions of *lhp1-6* and *lhp1-3* mutations. Region between SnaBI and SpeI sites used to generate transgenic *LHP1-eGFP* constructs. eGFP tag inserted at protein C-terminus. (B) Confocal microscope images of LHP1-eGFP proteins in Arabidopsis root meristems.

Berry_Fig_S4

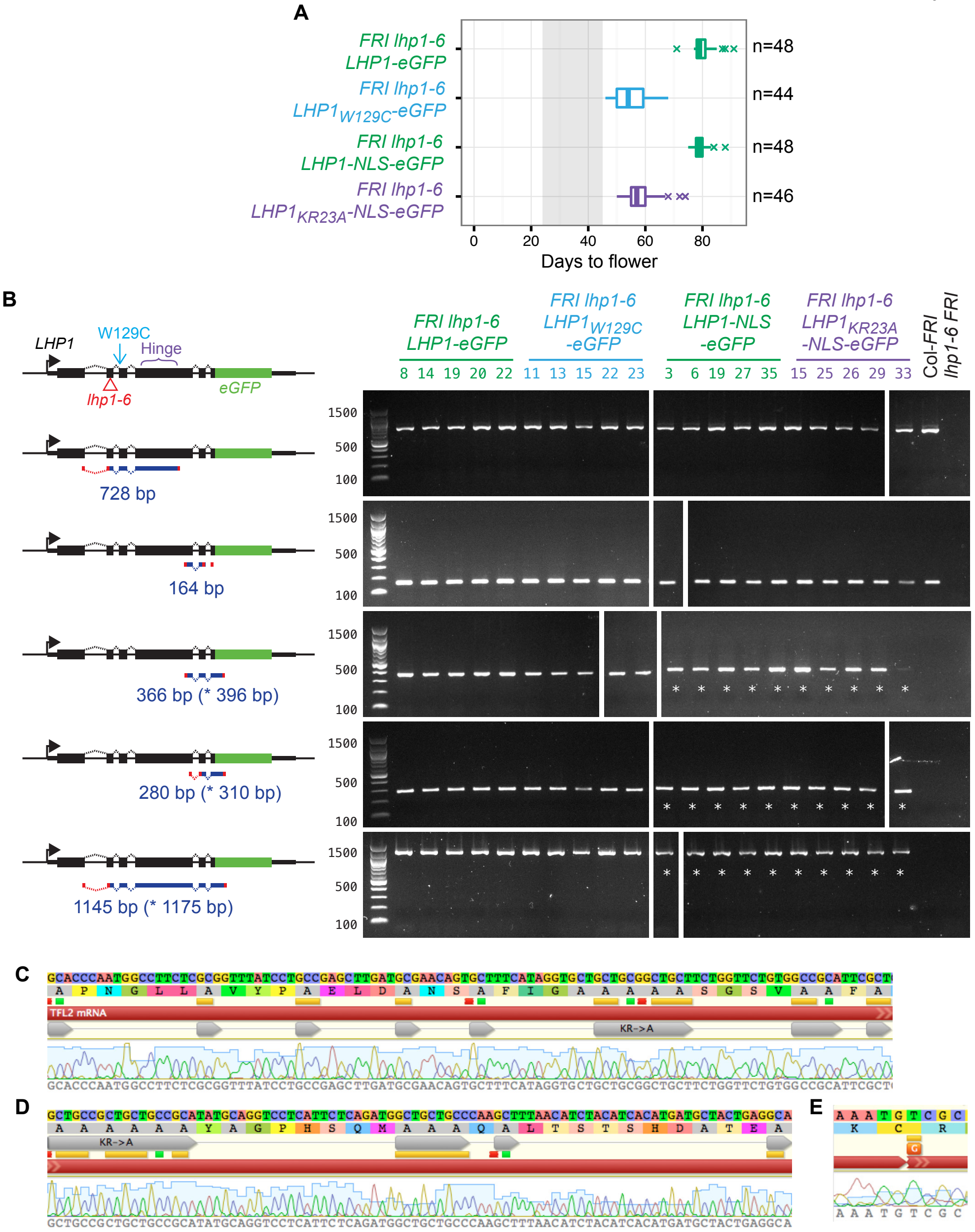

Supplemental Figure S4. Characterisation of *LHP1-eGFP* transgenic plants. (A) Flowering time for T1 generation *LHP1* transgenic lines (seed of transformed flowers). Plants were germinated on soil and sprayed with BASTA for selection. Grey box indicates 3-week cold treatment. *n* signifies number of independent transgenic lines retained. (B) RNA was extracted from plants expressing LHP1-eGFP and analysed by RT-PCR using amplicons indicated on left. Position of primers indicated in red on the amplicon. * indicates size for constructs containing NLS sequence. (C-D) Sequence confirmation of KR23A mutation in cDNA. Mutations from wild-type shown in grey. (E) Sequence confirmation of the W129C mutation in cDNA. Single G-T mutation from wild-type shown in orange.

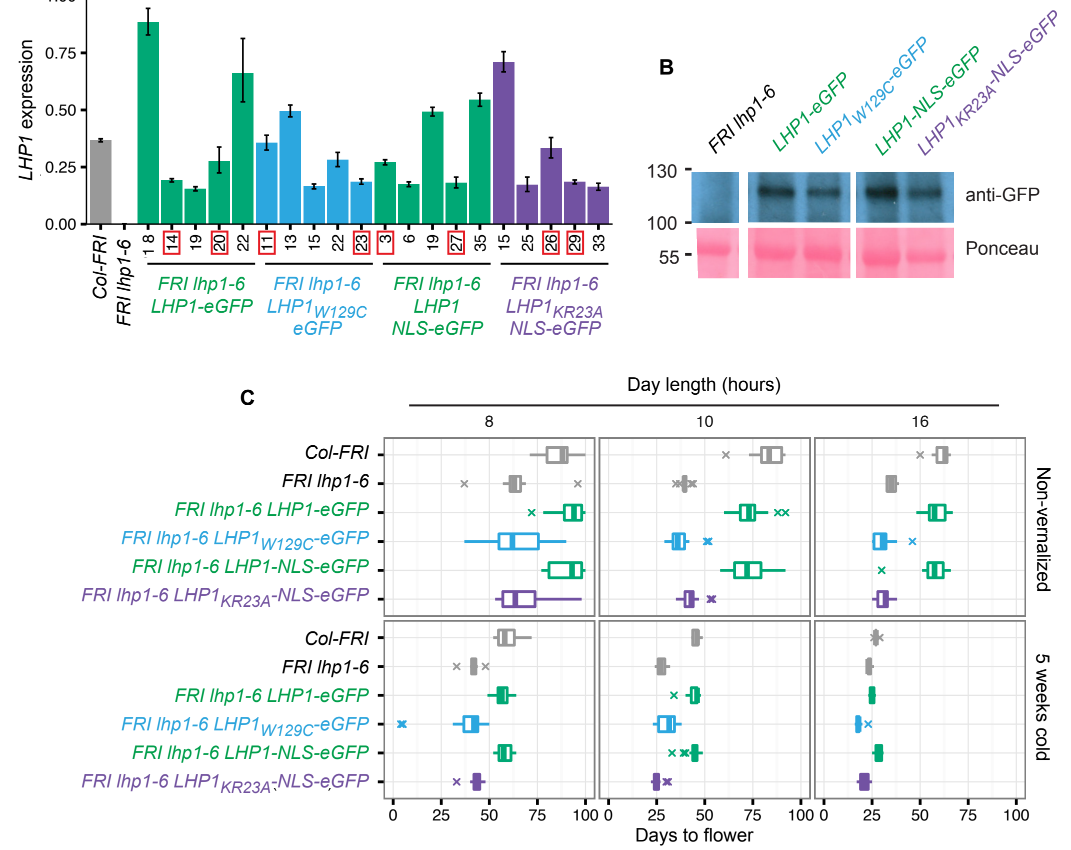

Supplemental Figure S5. Selection of *LHP1-eGFP* transgenic lines. (A) RT-qPCR comparing endogeneous *LHP1* expression in wild-type (Col-FRI) plants with expression of *LHP1* from transgenes in *FRI lhp1-6* background. Lines selected for further experiments shown in red boxes. (B) Anti-eGFP immunoblot using extract from transgenic *LHP1-eGFP* plants. (C) Flowering time for T3 generation *LHP1-eGFP* plants compared to wild-type (Col-*FRI*) and *FRI lhp1-6*. Top panels show results for warm growth conditions (22°C), while lower panels show results with a 5-week treatment at 4°C (not included in day count). Photoperiod indicated above. Results summarize 12 plants each for two independent lines (n=24).

Berry_Fig_S6

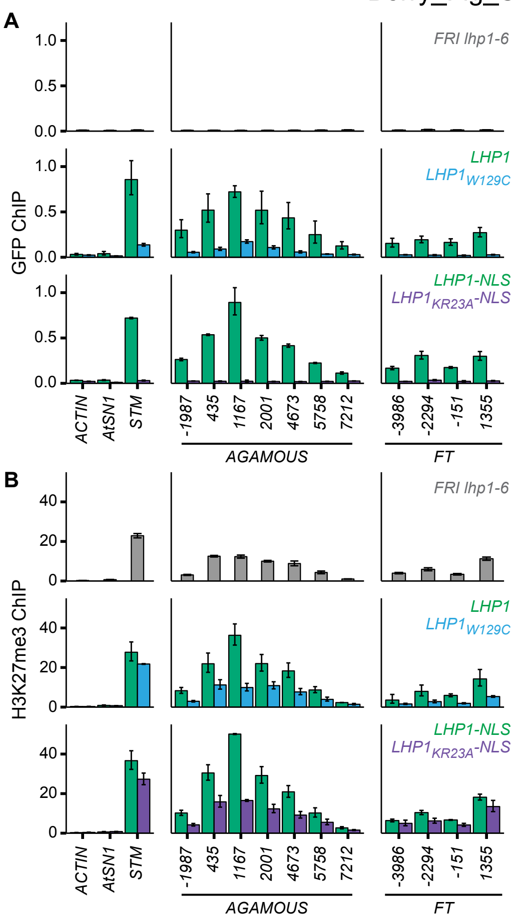

Supplemental Figure S6. Chromatin of Polycomb target genes in *LHP1* mutants. (A) LHP1 occupancy at *ACTIN, AtSN1, STM, AG* and *FT* as determined by anti-GFP ChIP-qPCR. Data represented as percentage of input DNA. Samples were taken 10 days after a 6-week cold treatment. All plants remained vegetative. Primer positions indicated for *AG* and *FT* relative to TSS. (B) H3K27me3 ChIP-qPCR represented as a percentage of H3 ChIP. In both cases, error bars represent s.e.m. (n=3).

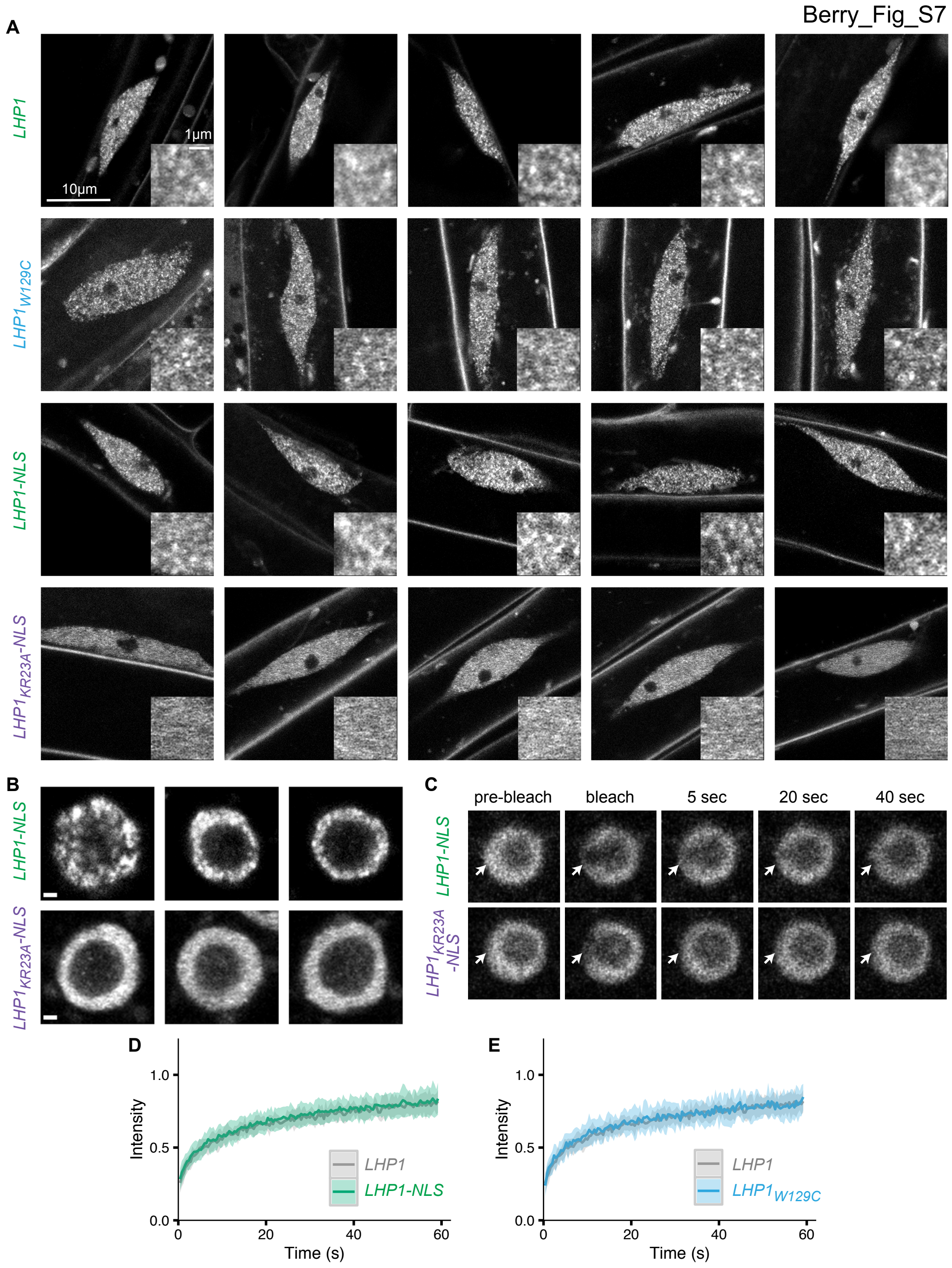

Supplemental Figure S7. Subnuclear distributions and dynamics of LHP1 mutants. (A) Subnuclear distribution of LHP1-eGFP fusion proteins in differentiated root epidermal cells. Inset panels show a zoom of the nucleus. All images have the same magnification, scale bars shown in top left only. (B) Subnuclear distribution in meristematic root epidermal cells. Scale bars are 1μm. (C) Representative FRAP images showing differences in recovery for LHP1-eGFP and $LHP1_{KR23A}$-eGFP in root epidermal cells. (D, E) Comparison of LHP1-eGFP

FRAP (n=10) with that of LHP1-NLS-eGFP (n=22) or $LHP1_{W129C}$-eGFP (n=15). Shaded regions around mean represent s.d.

Supplemental Table S1: PCR primer sequences

**Primers used for *FLC, AG, FT* ChIP**		
**Primer position**	**Sequence 5'-3'**	**Note**
FLC_-2320_F<br>FLC_2267_R	ATCCAGAAAAGGGCAAGGAG<br>CGAATCGATTGGGTGAATG	
FLC_-1599_F<br>FLC_-1530_R	TGGAGGGAACAACCTAATGC<br>TCATTGGACCAAACCAAACC	
FLC_-392_F<br>FLC_272_R	ACTATGTAGGCACGACTTTGGTAAC<br>TGCAGAAAGAACCTCCACTCTAC	
FLC_-49_F<br>FLC_53_R	GCCCGACGAAGAAAAAGTAG<br>TCCTCAGGTTTGGGTTCAAG	
FLC_157_F<br>FLC_314_R	CGACAAGTCACCTTCTCCAAA<br>AGGGGGAACAAATGAAAACC	
FLC_416_F<br>FLC_502_R	GGCGGATCTCTTGTTGTTTC<br>CTTCTTCACGACATTGTTCTTCC	
FLC_652_F<br>FLC_809_R	CGTGCTCGATGTTGTTGAGT<br>TCCCGTAAGTGCATTGCATA	
FLC_1144_F<br>FLC_1257_R	CCTTTTGCTGTACATAAACTGGTC<br>CCAAACTTCTTGATCCTTTTTACC	
FLC_1533_F<br>FLC_1670_R	TTGACAATCCACAACCTCAATC<br>TCAATTTCCTAGAGGCACCAA	
FLC_1933_F<br>FLC_2171_R	AGCCTTTTAGAACGTGGAACC<br>TCTTCCATAGAAGGAAGCGACT	
FLC_2465_F<br>FLC_2560_R	AGTTTGGCTTCCTCATACTTATGG<br>CAATGAACCTTGAGGACAAGG	
FLC_3197_F<br>FLC_3333_R	GGGGCTGCGTTTACATTTTA<br>GTGATAGCGCTGGCTTTGAT	
FLC_3998_F<br>FLC_4178_R	CTTTTTCATGGGCAGGATCA<br>TGACATTTGATCCCACAAGC	
FLC_4322_F<br>FLC_4469_R	AGAACAACCGTGCTGCTTTT<br>TGTGTGCAAGCTCGTTAAGC	
FLC_5139_F<br>FLC_5244_R	CCGGTTGTTGGACATAACTAGG<br>CCAAACCCAGACTTAACCAGAC	
FLC_6057_F<br>FLC_6175_R	CGTGTGAGAATTGCATCGAG<br>AAAAACGCGCAGAGAGAGAG	
FLC_6877_F<br>FLC_6947_R	TTGTAAAGTCCGATGGAGACG<br>ACTCGGCGAGAAAGTTTGTG	
AG_-2033_F<br>AG_-1941_R	AAAGCAACCTTCGATTGTGGTCCATC<br>GCTTTGGGTCAAGCTTTCATGGTCG	

AG_375_F<br>AG_494_R	ACCCATCTCTTCACCAGCACAACC<br>GGGGGAGAAGAACAAAGGGGAAAGTT	
AG_1094_F<br>AG_1239_R	TCTCCCTTGAGGAAATCTGG<br>GCGACTTCAGCATCACAAAG	
AG_1961_F<br>AG_2041_R	AGTTTTGGGAAACAAATTGGGGAGAGA<br>TGGAGGATGGATGATCACAAAACAGA	
AG_4621_F<br>AG_4724_R	AGGCAATTGATGGGTGAGACGATAGGG<br>ACCTTCTTGGATCGGATTCGGGT	
AG_5713_F<br>AG_5803_R	TCATGTGCTGAGTTCCATCCTCCT<br>GGTAGGCAGCTACAAAATGAACGAATG	
AG_7157_F<br>AG_7266_R	AGCGGTTTATGAGGTCGTTG<br>AATCGGGTTAGGCAGTAACG	
FT_-4115_F<br>FT_-3857_R	CAACGAGATTTGGGGTTAAG<br>TTGAATGCAGTCCGATTGTCC	5<br>(Adrian et al. 2010)
FT_-2454_F<br>FT_-2134_R	TGCATGCGAAAATCTAGTGG<br>GATGCATTGTTTAAGAAAATCAGG	6
FT_-251_F<br>FT_-51_R	GTGGCTACCAAGTGGGAGAT<br>TAACTCGGGTCGGTGAAATC	9<br>(Adrian et al. 2010)
FT_1212_F<br>FT_1497_R	GCTCAAACATGTTGCTCGAA<br>TGCGATCAGTAAAATACACAGACA	11<br>(Adrian et al. 2010)
**Control and reference genes for ChIP**		
ACTIN_F<br>ACTIN_R	GATATTCAGCCACTTGTCTGTG<br>CTTACACATGTACAACAAAGAAGG	
STM _F<br>STM _R	GCCCATCATGACATCACATC<br>GGGAACTACTTTGTTGGTGGTG	
AtSN1_F<br>AtSN1_R	CCAGAAATTCATCTTCTTTGGAAAAG<br>GCCCAGTGGTAAATCTCTCAGATAGA	
**Primers for RT-qPCR**		
FLC_F<br>FLC_R	AGCCAAGAAGACCGAACTCA<br>TTTGTCCAGCAGGTGACATC	*FLC* expression
AG_F<br>AG_R	CCGATCCAAGAAGAATGAGCTCTT<br>CATTTTCAGCTATCTTTGCACGAA	*AGAMOUS* expression<br>(Barrero et al. 2007)
AP1_F<br>AP1_R	CGACGTCAATACAAACTGGTCGAT<br>CTTTAGGGCTCATTGCTTGCA	*APETALA1* expression<br>(Barrero et al. 2007)
AP3_F<br>AP3_R	CCCTAACACCACAACGAAGGAGAT<br>GTTTCCTCTTGGTTTCTTGCATTC	*APETALA3* expression<br>(Barrero et al. 2007)
STM_F<br>STM_R	GAAGCTTACTGTGAAATGCTCG<br>AACCACTGTACTTGCGCAAGAG	*STM* expression<br>(Spinelli et al. 2011)
LHP1_F<br>LHP1_R	TGAGGAGTTGGACATCACGA<br>CTTCCCATCAGACCTCAGCG	*LHP1* expression

UBC _F<br>UBC _R	CTGCGACTCAGGGAATCTTCTAA<br>TTGTGCCATTGAATTGAACCC	For normalization<br>(Czechowski et al. 2005)

SUPPLEMENTAL REFERENCES

Adrian J, Farrona S, Reimer JJ, Albani MC, Coupland G, Turck F. 2010. cis-Regulatory elements and chromatin state coordinately control temporal and spatial expression of FLOWERING LOCUS T in Arabidopsis. *Plant Cell* **22**: 1425–1440.

Barrero JM, González-Bayón R, del Pozo JC, Ponce MR, Micol JL. 2007. INCURVATA2 encodes the catalytic subunit of DNA Polymerase alpha and interacts with genes involved in chromatin-mediated cellular memory in Arabidopsis thaliana. *Plant Cell* **19**: 2822–2838.

Czechowski T, Stitt M, Altmann T, Udvardi MK, Scheible W-R. 2005. Genome-wide identification and testing of superior reference genes for transcript normalization in Arabidopsis. *Plant Physiol* **139**: 5–17.

Fischle W, Wang Y, Jacobs SA, Kim Y, Allis CD, Khorasanizadeh S. 2003. Molecular basis for the discrimination of repressive methyl-lysine marks in histone H3 by Polycomb and HP1 chromodomains. *Genes Dev* **17**: 1870–1881.

Jones JDG, Shlumukov L, Carland F, English J, Scofield SR, Bishop GJ, Harrison K. 1992. Effective vectors for transformation, expression of heterologous genes, and assaying transposon excision in transgenic plants. *Transgen Res* **1**: 285–297.

Spinelli SV, Martin AP, Viola IL, Gonzalez DH, Palatnik JF. 2011. A mechanistic link between STM and CUC1 during Arabidopsis development. *Plant Physiol* **156**: 1894–1904.
